# An exploratory study to understand how people use Twitter to share experiences or information about spinal stenosis

**DOI:** 10.1186/s12998-022-00465-x

**Published:** 2022-12-28

**Authors:** Lillian L. C. Li, Arnold Y. L. Wong, Gregory N. Kawchuk

**Affiliations:** 1 Department of Rehabilitation Sciences, The Hong Kong Polytechnic University, Hung Hom, Hong Kong SAR China; 2 Department of Physical Therapy, University of Alberta, Edmonton, Canada

**Keywords:** Twitter, Health information sharing, Spinal stenosis, Physical and psychosocial wellbeing

## Abstract

**Background:**

Spinal stenosis is a narrowing of the spinal canal that may compress neurological tissues resulting in pain and disability. Although previous qualitative studies have solicited data regarding the life experience of patients with spinal stenosis or their opinions on relevant non-surgical treatments, their data was collected from participants in a controlled setting. Therefore, it remains unclear whether patients’ or caregivers’ concerns/opinions about spinal stenosis would be different in a non-experimental environment. Since Twitter is a popular online platform for people to share information and interact, it may reveal people’s thoughts and attitudes about spinal stenosis. This study aimed to identify tweets that are related to spinal stenosis on Twitter, and to categorize them into common themes.

**Methods:**

A social media monitoring and analysis software program (TalkWalker) was used to search relevant tweets using the keywords 'spinal stenosis' and 'stenosis' between 29 May 2019 and 24 June 2020. Two independent reviewers screened and conducted content analysis of the tweets and classified the tweets into different themes.

**Results:**

Of 510 identified tweets, 362 tweets met the selection criteria. Five themes were identified: (1) compromised physical, psychological, and social wellbeing (n = 173); (2) diverse treatment options (n = 69); (3) coping strategies (n = 30); (4) dissemination of scientific information (n = 86); and (5) health policy (n = 4). Most of the tweets revealed negative impacts of spinal stenosis on patients' physical and psychosocial wellbeing. People with spinal stenosis shared their experiences and sought helps from others, while some people used Twitter to disseminate relevant information and research findings.

**Conclusions:**

This is the first study exploring Twitter using an online analytical tool to identify themes related to spinal stenosis. The approach not only helps understand people’s concerns about spinal stenosis in an uncontrolled environment, but also can be adopted to monitor influences of diseases or public health education on Twitter users.

## Introduction

Spinal stenosis is a narrowing of the spinal canal that may compress the dural sac, spinal cord, or nerve roots [1–3], leading to various neurological signs and symptoms [4]. Spinal stenosis can be classified as cervical, thoracic, and lumbar spinal stenosis [5, 6]. While thoracic stenosis is relatively uncommon in adults, cervical and lumbar spinal stenosis are more prevalent. A cadaveric study found that cervical spine stenosis was present in 4.9% (23 out of 469 specimens), 6.8% (15 out of 220 specimens) and 9.0% (six out of 66 specimens) of donors aged 18 years or above, people aged 50 years or older, and people aged at least 70 years, respectively [5]. Likewise, lumbar spinal stenosis is common and is the leading cause of spinal surgery for patients aged over 65 years [7]. A recent systematic review revealed that the pooled prevalence of lumbar spinal stenosis based on radiological criteria was 38% in the general population (mean age: 53 years, range: 32–93 years), and 32% in patients from secondary care (mean age: 52 years, range: 19–95 years). Similarly, the mean prevalence of clinically diagnosed lumbar spinal stenosis was 11% in the general population (mean age: 62 years, range: 19–93 years), and 39% among patients from mixed primary and secondary care (mean age 65 years, range: 20–96 years) [8].

Depending on the severity of nerve root and/or cord compression, cervical spinal stenosis may cause neck pain and/or neurological signs (e.g., numbness, tingling or weaknesses in upper and lower limb muscles) [9], while lumbar spinal stenosis may cause neurogenic claudication after prolonged walking or standing [10]. Although these symptoms may be temporarily alleviated by changing positions (e.g., sitting or lumbar flexion in people with lumbar spinal stenosis) [11], persistent symptoms would compromise patients’ daily activities and increase their risk of falls [11]. Severe spinal stenosis may also cause bowel and/or bladder dysfunction. Conservative treatments (e.g., medications, supervised exercises, manual therapy, etc.) are the first-line treatments for patients with mild to moderate symptoms [12]. If symptoms worsen after 2–3 months of conservative treatments, surgical interventions may be considered [13].

Although prior qualitative research has attempted to understand the lived experience of patients with lumbar spinal stenosis or their opinions regarding conservative treatments for spinal stenosis, their findings were limited by collecting data from a small group of participants recruited from one or two clinics, or clinical trials [14–17]. Their findings might not include opinions from other stakeholders (e.g., caregivers). Further, these studies might be subject to selection or recall bias [14, 18, 19]. Therefore, it remains unclear whether patients’ or caregivers’ concerns/opinions about spinal stenosis may differ in a non-experimental environment. Given that it is not uncommon for some patients with chronic conditions (e.g., spinal stenosis) to share their experiences/frustrations on social media in real time, social media may be a potential new platform to solicit information from patients or caregivers regarding spinal stenosis that cannot be obtained from traditional methods.

Of various social media (Facebook, Instagram, Tik Tok, etc.), Twitter is a real-time microblogging platform for users to share and interact with one another. Unlike Facebook where most conversations/interactions are private, Twitter is an open platform that allows all users to view others’ tweets and connect with likeminded people. Tweets on Twitter can be shared (retweeted), commented on, or liked by others. Twitter had 290 million monthly active users globally in 2019 [20]. Twitter is deemed to be a low cost real world platform for researchers to estimate the impacts of diseases on different aspects of target or unselected populations [21, 22]. Additionally, Twitter provides a unique opportunity for researchers to communicate and cooperate across disciplines, as well as disseminate research findings globally [23, 24]. Information solicited from such a platform may guide future research [25, 26].

Given the above, it is conceivable that Twitter may contain useful information related to spinal stenosis that has never been explored. As such, the current study aimed to: (1) understand types of spinal stenosis-related information on Twitter; and (2) evaluate Twitter users’ perspectives regarding spinal stenosis.

## Methods

### Study design

A mixed method quantitative and qualitative content analysis was used to evaluate Tweets written in English.

### Talkwalker

A real-time social media monitoring and analysis software program, Talkwalker Quick Search (Talkwalker, Luxembourg, Luxembourg), was used to identify relevant tweets. Talkwalker can analyze activities and behaviors of anonymous target groups or users on various social media (Facebook, Twitter, Maipo, etc.), search for the most relevant information on pertinent topics, obtain relevant news from anonymous users, list the post time with the content's links, track brand images, identify emerging trends on social media in real-time, and use artificial intelligence algorithms to directly download the relevant information [27].

Tweets containing the keywords "stenosis" or "spinal stenosis" posted between May 29, 2019 and June 24, 2020 were identified by TalkWalker Quick Search (Talkwalker, Luxembourg, Luxembourg). The identified tweets also contained embedded information such as tweet types, original tweet links, the number of followers, retweets, etc. These were then downloaded as an Excel file. The related attributes of those searched tweets are listed in Table 1.

**Table 1:** Descriptions of properties of the extracted tweets

Properties	Descriptions
Matched profile	Searching for stenosis and/or spinal stenosis
Content snippet	Any tweets mention the search terms (i.e., stenosis, or spinal stenosis)
Original Twitter user's home page	A link to the homepage of the user who posted the tweet
Original tweet link	A link to the user's original tweet
Sentiment	The tweets that mentioned some simple or typical words about emotions (e.g., happy or sad)
Post type	The format of tweets (i.e., text, link, image, or video)
The type of sentence appears	The type of tweets (i.e., comment, new host, twitter reply, question)
Retweets	Number of tweets is retweeted by other users
Followers	Number of followers or subscribers
Data source	The location that the tweet was posted (i.e., region, country)
Gender	It shows the gender of a given Twitter user (i.e., female, male, unknown)
Word count	The number of words in a tweet

### Selection of eligible tweets

A reviewer (LL) screened for eligibility of the identified tweets. Tweets were included for analysis if they were written in English and were related to spinal stenosis or associated treatments in humans. For example, tweets concerning impacts of spinal stenosis on physical or psychosocial wellbeing (e.g., sports participation) of users or users’ friends were included. Other inclusion criteria included: treatments or coping strategies related to spinal stenosis; scientific literature related to spinal stenosis; or policies related to patients with spinal stenosis. Tweets were excluded if they: (1) only mentioned spinal stenosis among many other diseases; (2) described symptoms (e.g., lower limb numbness) that were unrelated to spinal stenosis; (3) were advertisements, marketing campaigns, or identical tweets posted by different user accounts.

For tweets with ambiguous contents, the original tweets and relevant tweet sources/websites were browsed. A second reviewer (AW) counterchecked the content independently. Any disagreements between the two reviewers were resolved by discussion. Persistent disagreements were planned to be resolved by consulting a third reviewer (GK), but it was not needed in the current study. Twitter users who posted those tweets were not contacted.

### Analysis of the included tweets

The number of “likes” per tweet, and the overall engagement rate of all included tweets (i.e., the total number of people liked, retweeted, or gave comments to the included tweets after viewing them) were retrieved from Talkwalker. Additionally, the current study adopted a six-step thematic analysis to analyze the tweets and classify different tweets into themes [28]. The six steps include: (1) data familiarization; (2) generating initial codes; (3) searching potential themes; (4) reviewing themes; (5) determining theme names, and (6) finalizing the result [28]. A reviewer (LL) analyzed the contents of the included tweets, evaluated the interactions among users (e.g., hosts and followers). A user who follows another user account is defined as a follower, and the original tweets published by a host will appear in the “Home” timeline of the follower. The reviewer (LL) also reviewed the links posted in the tweets to determine major content themes. The reviewer first familiarized herself with 50% of the included tweets to generate the initial codes and respective definitions in a codebook, and searched for potential themes. The codes and themes were continuously reviewed and refined until all tweets were analyzed. The codebook was used to categorize relevant tweets into a specific theme. Some tweets could be categorized into more than one code. Each tweet was evaluated in an iterative manner to ensure consistent coding into different themes. A second reviewer (AW) independently verified the classification of tweets and themes.

## Results

A total of 510 tweets were identified from Talkwalker. One hundred and ninety-one tweets were excluded. Specifically, 84 tweets only mentioned the term “spinal stenosis”, 15 tweets were unrelated to spinal stenosis, and 49 tweets were advertisements (e.g., promotion of products or medical doctors). Therefore, 362 tweets from five continents (North America = 306; Europe = 35; Asia = 15; Africa = 4; Australia = 2) were used for data analysis. The number of "likes" per tweet ranged from 0 to 419 (the average number of "like" was 8.34; 159 tweets was not “liked” by anyone). The engagement rate of the 362 tweets was 0.02% (3,861 engagements out of 22,293,674 views). Three types of Twitter users were identified from the included tweets: (1) patients with spinal stenosis who shared their stories, feelings, and treatment experiences; (2) users who commented on other people with spinal stenosis (e.g., athletes, relatives, or friends); and (3) clinicians or researchers who disseminated the latest research findings regarding spinal stenosis.

### Main themes of tweets

From 362 included tweets, five themes were identified: (1) physical and psychosocial impacts of spinal stenosis (n = 173); (2) treatments for spinal stenosis (including medications, injection, and surgeries) (n = 69); (3) seeking help from others (n = 30); (4) evidence-based information from research or scientific information (n = 86); and (5) health policy (n = 4) (Table 2).

**Table 2:** Themes and subthemes of the included tweets

Main theme	Subtheme	Description of tweets	Number of tweets
Physical and psychosocial impacts of spinal stenosis	Physical impacts	Symptoms, physical function, or balance	87
	Psychological impacts	Impacts on emotion and feelings	50
	Impacts on social roles	Work or life	36
Treatments	Medication	Medications	32
	Surgery	Various spine surgeries	24
	Injection	Spinal injections or its effect on users	2
	Other treatments	Other non-pharamceutical treatments for spinal stenosis	11
Seeking help from others	Seeking help	Asking for donation, tips, and suggested medications related to spinal stenosis	22
	Prayers	Prayers for the situation, or asking other people to pray for their recovery	8
Evidence-based information/ scientific information	Not applicable	The latest research update or research results or scientific information about spinal stenosis	86
Health policy	Not applicable	Tweets related to relevant policy	4

### Theme 1: physical and psychosocial impacts of spinal stenosis

#### Physical impacts

Table 3 lists some representative samples of tweets that were related to physical impacts of spinal stenosis on Twitter users, or their family or friends. Pain was the most frequently mentioned symptom in patients' tweets (49 tweets). Pain was reported at different body parts, including lower back and legs: *"…I suffer from severe spinal stenosis … I'm in pain 24/7 even on very strong pain meds and nerve meds …"* Many people with spinal stenosis also expressed difficulty in walking, which led to altered walking patterns (e.g., a waddling gait) or becoming wheelchair bound (16 tweets; Table 3). Importantly, some of them reported having experienced falls:*"…I have spinal stenosis and it affects my mobility and cause me to fall …."* Additionally, some users complained about reduced body height (Table 3).

**Table 3:** Tweets related to physical impacts of spinal stenosis

Types	Representative tweets	Counts
Pain	*"…my husband whose in horrific untreated pain due to severe spinal stenosis…."* *"…I suffer from severe spinal stenosis … I'm in pain 24/7 even on very strong pain meds and nerve meds …"* *“I have fibromyalgia type pain stemming from spinal stenosis and hypermobility joint syndrome. I love to crochet and craft (never was good at drawing) but it's the same feeling of "there was nothing and now there's something pretty cool" that makes me happy,”* *“I have an upcoming ACDF d/t C5/6 radiculopathy and spinal stenosis. I have never felt nerve pain before and I now know how hard it is to describe.”* *“My health I have chronic pain from spinal stenosis and a limp from my spina bifida. Not a day goes by some part of my body isn't hurting.* * It's hard to keep my spirits up when that is my daily lot. Self-pity is also a problem (as you may be able to tell) I fight against that.”*	50
Difficulty in walking or altered gait patterns	*"My ma can't walk more than a few feet at a time due to spinal stenosis, and I can't afford her surgery to get this fixed… "* *"I cannot walk due to spinal stenosis and nerve damage in my legs…"* *"Due to his abnormal bone growth, Tyrion has a narrowing and compression of his spinal cord (spinal stenosis). This causes a great deal of pain, especially in his lower back and legs; his characteristic waddling gait; and numbness in his lower extremities."* *"I don't know your condition, but I was in a wheelchair for a period due to spinal stenosis so I feel your sorrow…"*	16
Numbness	*"…I had issues from stenosis in c7 spinal cord causing numbness down my right arm."* *" Pretty sure my spinal stenosis is the cause of numbness and Pain…"*	8
Falls	*"…I have spinal stenosis and it affects my mobility and cause me to fall so having one of these could be priceless if I have an accident and I need help."*	7
Paralysis	*"I grew up poor and know how hard life is having a mental illness, kidney disease and paralyzed from thoracic spinal stenosis… "*	2
Other illness	*" I had a fever due to a spinal stenosis swelling condition not related to any virus. "*	1
Height	*"…I have spinal stenosis and that contributes to my loss of height."* *“I'm short and getting shorter. Here is my view on standing on a platform to be equal. l would bring a saws all, 2 make the podium shorter. Once just under 5′9"/ spinal stenosis and a twisted spine, now has me at 5′5."*	3

#### Psychological impacts

Fifty included tweets were related to the impacts of spinal stenosis on the Twitter users’ emotion. Table 4 lists some representative tweets from the first- and third-person perspectives. Most tweets indicated negative impacts of spinal stenosis on their psychological make up. Depression, anxiety, and other negative emotions were mentioned in multiple tweets related to spinal stenosis (Table 4). Importantly, one tweet mentioned that the user was having severe depression because of spinal pain and understood why others turned to the street or suicide. Another user tweeted that he/she attempted to seek help for suicide prevention because of spinal stenosis. Conversely, two tweets expressed some positive emotions despite spinal stenosis: *“I was born with it (spinal stenosis) and I’m rather lucky that I did not do anything to make it worse. I played HS football.. baseball.. never knowing how bad my spine was.. so I consider myself very lucky…”*.

**Table 4:** Tweets related to impacts of spinal stenosis on the psychology or social aspects of people

Types	Representative tweets	Counts
Psychological impacts		
Mental		
Depression	*"… I’m 26 have chronic pain from spinal stenosis can’t walk very well and my depression has been so much worse I can’t get that 1200 check cause I’ve been unable to work and I’m off my meds cash app is $basuraboye.”*	5
Anxiety	*"… Spinal Stenosis, and Diabetes contribute heavily to my anxiety and mentally illness/sheer terror…."*	3
Self -pity	*"…My health I have chronic pain from spinal stenosis…Self-pity is also a problem (as you may be able to tell) I fight against that. "*	1
First-person perspective		
Negative emotion	*"I have it, (spinal stenosis), and take it from me, it hurts like a bitch… "* *"…My intense AGONY from Spinal Stenosis began on that trip & because I wasn't able to leave the room for 10 of the 14 vacation days, it would have been TORTURE! "* *“@tinyneondemon Sweetie I feel ya! I have spinal stenosis and degenerative disc disease and some days I can’t move at all* *I am scared to death of surgery.”* *“I suffer from spondolothesis, stenosis, scoliosis, arthritis, a fracture, and a cracked tailbone and suffer every single day in terrible pain bc I am so under medicated. I am now in the midst of another severe depression. I can seriously see why ppl turn to the street or suicide.”* *“My spinal chord is being crushed by the stenosis. Suicide prevention won't help me. I tried to get in to get help. I can get scripts, but can't get them filled anymore. My pharmacy just told me to go somewhere else. All the others told me they can't take new opiate patients.“*	25
Positive emotion	*" …I was born with it (spinal stenosis) and I’m rather lucky that I did not do anything to make it worse.. I played HS football.. baseball.. never knowing how bad my spine was.. so I consider myself very lucky… plus I was in the military.. keep going strong bud."*	2
Third-person perspective		
Negative emotion	*"…Wright's Spinal Stenosis should be a 1 seed. Absolutely devastating… "* *" I’m sad we never got a season of wright and Alonso together. Damn imagine if he never got spinal stenosis. "* *"…I knew of a man who was taken off all of his opioids. He had spinal stenosis and was miserable. His wife left him. He kids refuse to talk to him. He said the only reason why he didn't commit suicide was because of his dog. I cried…"*	7
Positive emotion	*"I worked with a woman who had spinal stenosis. She was one of my best friends and in constant pain. Alan Dorman is making a large sacrifice. I admire and appreciate him. Praying for him and his surgical team."*	7
Impacts on social roles		
Difficulty in voting	*"I am an American Veteran with spinal stenosis and cannot stand in long lines, so Americans should also be able to vote by mail and have their ballots counted! "*	1
Difficulty in working	*"…I have been sick and now have severe spinal stenosis, have not been able to work…"* *"…I have spinal stenosis/osteoarthritis of the spine, making it hard for me to work."* “*This was perfect for me because I have acute spinal stenosis and I was able to work in spurts and rest my back as needed. I can’t sit or stand or stay in one position for very long without being in pain. This allowed me to work at my own pace.”*	15
Not being able to participate in sports	*"DeGabriel Floyd is handling this as well as you can expect. Doctors diagnosed the UT linebacker with spinal stenosis and missed the entire season this year…"* *"Sting had spinal stenosis before that match… he just didn’t know it… and like Sting said himself…. “Seth did not end my career… I was already messed up… I just wasn’t aware”* *"Stone Cold also has the same spinal stenosis which ended his career. "*	18
Affecting relationship	*"…my husband whose in horrific untreated pain due to severe spinal stenosis. I can't even hug him without hurting him if I squeeze too hard. Intimacy is not an option for either of us"*	2

#### Impacts on social roles

Spinal stenosis affects patients’ social roles. As it is difficult for some patients with spinal stenosis to stand for a prolonged period, one Twitter user complained that he could not cast his vote in person because he could not stand in a queue. Further, some patients with spinal stenosis could not work, while others were fired, ended their careers, or were forced to retire (33 tweets) (Table 4). For instance, 18 tweets were related to athletes in professional baseball (n = 7), American football (n = 5), wrestling (n = 5), and basketball teams (n = 1) who were forced to stop their practice or careers due to spinal stenosis.

### Theme 2: treatments for spinal stenosis

Different treatments for spinal stenosis (e.g., medications, surgeries, injections, and physiotherapy) were mentioned in the included tweets (Table 5). Kratom was the most frequently mentioned medication. Specifically, 16 tweets from multiple Twitter accounts stated that Kratom reduced 75 percent of patients' pain. However, given the similarity of these tweets, the finding should be interpreted with caution because these tweets might be originated from spam accounts or people sponsored by a company. Overall, all spinal stenosis-related medications posted on Twitter were about painkillers. Spine surgery was the second most mentioned treatment in the included tweets. Diverse surgical approaches (e.g., radio ablation, anterior cervical discectomy and fusion, or lumbar decompression) were mentioned although some tweets did not specify the type of surgery (Table 5). Some Twitter users reported improvements after surgery: *"Dr. D’Ariano has brought me back to being virtually pain free 6 weeks out of surgery. I’m a 73-year-old women who had severe spinal stenosis, 3 herniated lumbar discs, and so forth!"* Others reported negative post-surgical results: *"…I had spinal stenosis this cut the exercise regime as I couldn't walk better after surgery a year ago…"* (Table 5).

**Table 5:** Tweets related to treatments of spinal stenosis

Medication	Effects	Representative tweets	Counts
*Medications*			
Kratom	Positive	*"…Kratom relieves 75 percent my pain from fibromyalgia, arthritis, spinal stenosis, frayed meniscus, sciatica, shoulder tendinitis *etc*. With FDA approved medications I was bedridden using a walker and wheelchair. Taking kratom for pain I bike several or walk a couple miles daily…"* *"…Kratom relieves 75% of my pain from fibromyalgia, arthritis, spinal stenosis, frayed meniscus, shoulder tendinitis, and sciatica. Taking FDA approved medications I was bedridden using a walker and wheelchair 8 years. I now walk or bike miles daily. I even rock climb"*	16*
Opioid	Positive	*"NSAIDs won't help my severe spinal stenosis though and small doses of opioids do. "*	1
	Not mentioned	*"…I was on the same dosage for years (almost 10) …"*	1
Lyrica	Positive	*"…I thought Fibro and Spinal stenosis was bad but you really have it rough. Unfortunately CBD doesn't seem to work for me, the only thing that helps me is Lyrica…."*	1
X39	Positive	*"…Spinal stenosis is improving & pain reduce 90%…"*	1
Tetrahydrocannabinol	Positive	*" I'm right there with ya buddy (in texas) I have spinal stenosis so most days are hard on me and boy a little THC takes the pain away real quick."*	1
Enbrel	Positive	*"Jamie Meade has struggled with rheumatoid arthritis and spinal stenosis for 15 years…and he says Enbrel is a miracle drug…a miracle drug…"*	1
Pregabalin + Gabapentin	Negative	*" Gaba/Pregabalin- They are not effective for low back pain, sciatica, spinal stenosis…"*	1
Pregabalin	Negative	*"It would be great if pregabalin helped patients with low back pain, sciatica and spinal stenosis. It doesn’t. "*	1
*Cannabidiol* (CBD)	Negative	*"…I thought Fibro and Spinal stenosis was bad but you really have it rough. Unfortunately CBD doesn't seem to work for me…"*	1
Non-steroid anti-inflammatory drugs	Negative	*"…NSAIDs won't help my severe spinal stenosis though and small doses of opioids do. It's a balance…."*	1
Tramadol	Negative	*"…Tramadol helps a little during daytime. Not at night."*	1
Suboxone and Percocet	Not mentioned	*"…I get 6 mg of Suboxone and 20 whole Percocet to combat this with… "*	1
Oxycodone	Not mentioned	*"…I've got cervical spinal stenosis and they had me on 120 oxycoton a month…"*	2
Ibuprofen + Meloxicam	Not mentioned	*"I've been prescribed ibuprofen 800 mg for years 10 plus. With opiate pain killers.. Gave up the opiates 10 months ago,rely on meloxicam / IB profen800mg.. What do we do to control spinal stenosis bulging discs and disc disease?? "*	1
*Surgery*			
Just mentioned surgeries without providing specific details	Positive	*"Dr. D’Ariano has brought me back to being virtually pain free 6 weeks out of surgery. I’m a 73 year old women who had severe spinal stenosis, 3 herniated lumbar discs, and so forth!"* *"…after a few surgeries I can stand again…wheelchair to get back on my feet…"*	6
	Negative	*"I am not fake I suffer from severe spinal stenosis my bones around my spinal cord are choking my spinal cord and nerves. I'm waiting to have my 10th back surgery I'm in pain 24/7 even on very strong pain meds and nerve meds. I can't lose my insurance."* *"…I had spinal stenosis this cut the exercise regime as I couldn't walk better after surgery a year ago…"*	6
	Not mentioned	*"First symptom was foot drop and tripping. Took over a year for dx; they told her it was spinal stenosis and she had back surgery."*	3
SpineJack implants	Positive	*"…reduction of the retropulsed fragment, and improved spinal canal stenosis…"*	1
Radiofrequency ablation	Negative	*"I am 67 yrs old, I have a failed knee replacement, failed (3 each) spinal stenosis collapsed c5&6 with a bone spur. I have had radio ablation on my lumbar and neck no effect. "*	1
Anterior cervical discectomy and fusion (ACDF)	Negative	*"The ACDF, didn’t take away the pain. I have congenital cervical/spinal stenosis, on top of an unstable spine full of bone spurs/osteoarthritis/and herniated Discs…"*	1
ACDF at C5/6	Not mentioned	*"I have an upcoming ACDF at C5/6 radiculopathy and spinal stenosis…"*	1
Spinal fusion neck surgery	Not mentioned	*"…He had spinal fusion neck surgery, and that should have been the end…"*	1
Lower lumbar spinal fusion	Not mentioned	*"I just had lower lumbar spinal fusion this past July, due to 6 months of spinal stenosis and nerve pain…"*	1
Lumbar decompression	Not mentioned	*"…I had a MRI and eventually a lumbar decompression…"*	1
Necessity of surgery	Not mentioned	*“I have spinal stenosis and facet joint arthritis…Eventually I'll probably need surgery like my aunts both did.”*	3
*Injection*			
A transforaminal epidural steroid injection (TFESI)	Positive	*"…I've been able to manage the pain with TFESI…"*	1
Steroids and lidocaine	Negative	*" When I used to get steroids and lidocaine injected into my neck, only made it worse!…Had spinal stenosis…"*	1
*Other treatments*			
Tempur-Pedic Neck Pillows	Positive	*"Max, if you are willing to spend the money the Tempur-Pedic Neck Pillows. I have profound spinal stenosis and for years had a hard time finding a pillow that could provide both support and comfort. My neurosurgeon recommended this pillow. It's amazing and worth the extra $$."*	1
Mattress	Not mentioned	*"I also have fibro and spinal stenosis. I have researched a lot and have decided to get a Saatva hybrid mattress with an adjustable base. I watched a lot of YouTube reviews.*"	1
Custom orthotics	Positive	*"Custom orthotics help relieve pain and improve motion for patients with sciatica, spinal stenosis, and low back pain!…"*	1
Transcutaneous electrical neurostimulation	Positive	*"…I had back surgery for spinal stenosis. I understand nerve pain. Done it all. My back pain is manageable now thank God and usually not an issue. I bought a BioWave. Helps a lot."*	1
	Negative	*" I have spinal stenosis, sciatic in both legs and the fibromyalgia. It sucks. I have bad neuropathy many nights.Oh, I have a TENS, doesn't help…"*	
Hot water	Positive	*"… Good for spinal stenosis to have hot water jets hitting that area. Have a relaxing and healing time! "*	1
Hot tub	Negative	*"Once upon a time I was 5′9", and then spinal stenosis caused my spine to twist and I am only 5′6" tall, and shrinking. Staying out of the hot tub has helped a little!…"*	1
Physiotherapy	Positive	*"I've had 8 surgeries for osteophytes and stenosis. Get a good PT. Try manual traction, maybe a traction unit if it's spinal or thoracic. Do chin tucks, gentle isometric exercise and stretching but nothing that hurts. Get in a pool if you can, i swim high * * for the pain. "*	1
Massage	Positive	*"I have a history of 3 car accidents, spinal stenosis, herniated disks, piriformis syndrome and on…I have therapeutic massage regularly too. Back pain relief is a process."*	1
Working on the farm	Positive	*"I have spinal stenosis in my neck and spine but I've found that the movement of working on the farm has helped me tremendously I just take it slow and steady and use things to make it easier. Better than physical therapy ever did."*	1
Stretching, walking, and ice therapy	Positive:	*"…But now I have spinal stenosis. I found stretching, walking and ice the best treatment. "*	1
Artisan plant	Positive	*"…Been a long day and my shoulder tendonitis is acting up along with my fibromyalgia, arthritis, spinal stenosis and sciatica. So thankful not take pain meds anymore. * *Artisan Botanicals "*	1

^*^All tweets started with a sentence stating that Kratom relieves 75% of pain (n = 16). The second half of the tweets might mention not using a walker and a wheelchair (n = 1); or able to walk or bike, even rock climbing (n = 2); bike or walk (n = 1); or rock climbing (n = 1)

Other treatments or self-management methods were posted on Twitter. These interventions including specific mattresses, pillows, or conservative treatments (e.g., transcutaneous electrical neurostimulation, hot tub, hot water jets, and massage) (Table 5). Additionally, yoga, farm work, and stretching have also been mentioned for managing spinal stenosis (Table 5).

### Theme 3: seeking assistance from others

Given the negative impacts of spinal stenosis on various facets of patients’ life, people with spinal stenosis sought supports or assistance from other Twitter users (Table 6). Eleven tweets sought financial help from other Twitter users. Nine users asked for medical information related to spinal stenosis. Some users asked for prayers (n = 4) or social support (n = 4).

**Table 6:** Seeking helps from others through Twitter

Content	Tweets	Counts
*Seeking help*		
Money	*"Need help with medical bills waiting for 10th back surgery for severe spinal stenosis and revision of spinal cord stimulator my out of pocket is over $10 k but if I can come up with half we can go forward with my surgery my pain is 24/7…… please help! "*	11
Medications	*"…I got SSDI because I’d spinal stenosis, DDD and anxiety. Can u get ahold of some kratom? "*	2
Information	*"…I’m in pain 99% of the time. (Arthritis, spinal stenosis, Trigeminal neuralgia) got any tips? " "…I am a CPP, spinal stenosis, 4 discs herniated, Peripheral Neuropathy and severe Nerve Damage (just to name a few). I no longer have a Dr or the life I used to love. I stay home. please someone send me in the right direction. I can't take it!! " "Is there any literature specifically on the effects of squats and/or deadlifts in non-surgical spinal stenosis patients? "*	9
Social support	*"thank you for the wonderfully strong and beautiful greeting for today. May I ask a favor? I am having back surgery tomorrow. (Spinal stenosis). Would appreciate thoughts and prayers…"*	4
Prayers	*"…I've been praying for her and recently she has been feeling great. God is good! "* *"…May I ask a favor? I am having back surgery tomorrow. (Spinal stenosis). Would appreciate thoughts and prayers…"* *"I worked with a woman who had spinal stenosis. She was one of my best friends and in constant pain. Alan Dorman is making a large sacrifice. I admire and appreciate him. Praying for him and his surgical team."*	4

### Theme 4: evidence-based or scientific information

Table 7 lists six types of tweets related to scientific information of spinal stenosis (83 tweets). They included hyperlinks to journal articles, websites, PowerPoint files, YouTube videos, or discussions. Some tweets were linked to comprehensive overviews of spinal stenosis (including definition, pathology, diagnosis, medication, prevalence, nonsurgical and surgical treatments, surgical procedures, rehabilitation, and the risk of surgery). Twenty-four tweets were related to relevant journal articles (e.g., systematic reviews, meta-analyses, case-control studies, clinical trials, or validation of some spinal stenosis questionnaires). Another 24 tweets shared information or hyperlinks of different online articles regarding the pathology or development of spinal stenosis, and treatments for spinal stenosis. Six tweets shared hyperlinks of relevant YouTube videos or PowerPoint slides (Table 7).

**Table 7:** Dissemination of scientific information related to spinal stenosis by Twitter users

Types	Tweets	Counts
Clinical findings	*"Unstable traumatic L1 burst fx involving the lamina with retropulsion. SpineJack implants and cement injection. Impressive height restoration, reduction of the retropulsed fragment, and improved spinal canal stenosis. Resolved neuropathy in POD 1! "*	28
Journal articles	*"The factors of deterioration in long-term clinical course of lumbar spinal canal stenosis after successful conservative treatment "*	24
Articles on medical websites	*"Discover the causes and symptoms of spinal stenosis and pain management remedies you can use at home. *http://bit.ly/2VzatFa* "*	25
Links to PowerPoint presentations	*"Spinal stenosis happens over time and can affect your mobility, comfort, and how your bladder and bowel work. More information: *https://wb.md/2A5pR5K*"*	7
YouTube videos	*"Spinal Stenosis: Cause and Correction youtube/XwgmzhuTev8"*	3
Academic discussion	*"You can't have the symptoms and not have spinal stenosis. Check out Postural Restoration in Lincoln Nebraska. It's about rotation and compression"*	3

### Theme 5: health policy

Four tweets mentioned medical benefits, and three were related to asking the then U.S. President, Mr. Donald Trump, to sign the Emergency Funding Bill to mitigate the impacts of spinal stenosis on their lives: *"President Trump, please get congress to pass and you sign "The Emergency Money for The People Act" sponsored by Tim Ryan and others, I am disabled, and can't work due to a neck surgery and spinal stenosis. but I am not yet receiving SSI or SSDI."*

## Discussion

This is the first study to evaluate the impacts of spinal stenosis on people through the content analysis of tweets. Twitter users repeatedly mentioned the negative impacts of spinal stenosis on their physical and psychosocial wellbeing. Some patients even tweeted suicidal thoughts because of spinal stenosis-related pain. Our results reveal that Twitter is a broadcasting and communication platform for laypersons to solicit information related to spinal stenosis, or for academics/healthcare professionals to disseminate research findings or credible medical information regarding spinal stenosis and related treatments [29, 30]. Although users can garner relevant information on Twitter, they should be cautious and not automatically accept the information as being credible. For example, 16 very similar tweets stated that kratom was a drug approved by the Food and Drug Administration that reduced pain originated from spinal stenosis or other diseases by 75% (Table 5). However, the US Food and Drug Administration in fact warns people not to use kratom because it contains psychoactive compounds that are similar to opioids and may increase the risk of addiction [31]. Therefore, information gathered from Twitter should perhaps be better used as a discussion starter with appropriately informed healthcare providers.

The analysis of tweets allows researchers and clinicians to understand the diverse feelings or thoughts of Twitter users in a non-experimental setting. Unlike structured patient experience surveys that usually ask patients about their prior healthcare experiences after some time [32], Twitter users can tweet their thoughts, feelings, or experiences about treatments or diseases at anytime, or from anywhere. Some of these tweets may have less recall bias. In fact, sentiment analysis of social media has long been adopted to understand people’s opinions or feelings about services, events, or products [33–36]. Therefore, the tweet analysis may provide a new avenue to understand the concerns and thoughts of target populations, which may not be obtained from traditional surveys. These findings may help identify research gaps and inform future research directions. This approach may be very useful for diseases with low prevalence or for situation where researchers have difficult in soliciting opinions (e.g., under a pandemic situation).

Prior research has shown that patients with spinal stenosis have a poor quality of life, lower participation in daily activities, and limited ability to stay at work [37, 38]. The job satisfaction of patients with lumbar spinal stenosis is known to be significantly lower than healthy individuals [39]. Our findings concurred that Twitter users frequently mentioned negative impacts of spinal stenosis on work (e.g., inability to work, being fired, and early retirement). Some Twitter users also tweeted that certain elite athletes needed to suspend their usual practice or even end their career because of spinal stenosis. This finding agrees with prior research that athletes with spinal stenosis have a shorter career length than their healthy counterparts [40, 41]. Given the potential negative physical and psychological consequences of spinal stenosis, proper patient education is warranted to meet these patients’ desire for understanding the pathology, self-management techniques, conservative and surgical treatments, which can help inform their decision-making [14, 42].

Pain was the most common symptom in patients' tweets. Pain is the common reason for people with spinal stenosis to have decreased mobility and social participation [43]. Alarmingly, our analysis revealed that two patients had indicated potential suicidal thoughts because of pain, which has not been mentioned in previous research. Although speculative, this finding may be attributed to depression. It is known that approximately 20% of patients with lumbar spinal stenosis have clinically significant depression [44]. Many Twitter users also mentioned depression and other negative emotions in their tweets. It is possible that chronic pain, poor life satisfaction, and difficulties in coping with spinal stenosis may increase the risk of developing depression in patients with spinal stenosis [44–46]. Since concomitant spinal stenosis and depression can jeopardize patient's psychological and physical wellbeing in the long run even after spine surgery [46–48], future studies should investigate the effectiveness of multimodal approach in managing patients with concurrent spinal stenosis and depression. Clinicians should also be aware of mental health problems in these patients so as to provide timely management/referral.

Many patients with spinal stenosis seek relevant medical information through Twitter because they desire to have reassurance or helpful information (e.g., treatment options) for their conditions [49–51]. Patients with spinal stenosis or other diseases often seek help from relatives and friends to deal with their stresses and concerns [52]. The COVID-19 outbreak and social isolation that came with it, negatively, affected many patients with chronic diseases [53, 54]. Social media has become an essential platform for the public to access information and communicate with others instantly [55, 56]. Our results lend support to the notion that people with diseases want to learn from other patients' personal experiences, including treatment experiences [57]. Additionally, some Twitter users used hashtags to draw attention and connect with likeminded people. Our findings highlight that Twitter is an instant and inclusive online platform that allows users to proactively get involved in their inquiry process [58] and/or to express their feelings, or curiosity.

Additionally, some people with spinal stenosis used Twitter to seek prayers, social support, attention, and welfare. Financial need was the most common reason for people with spinal stenosis to seek help on Twitter. Prior research has reported that spinal stenosis may lead to job interruption, and high medical and hospitalization expenses, which may impose heavy economic burdens on patients and their families [59, 60]. Our findings concur that some patients with spinal stenosis may face high treatment costs, require frequent medical visits, and need to seek financial supports. Although our results cannot be generalized to all patients with spinal stenosis, it underscores the importance of proper social welfare support, reasonable healthcare costs, effective diagnosis and treatments, as well as sharing decision making for patients with spinal stenosis [10, 60–65].

The current study shows that Twitter can be used to provide relevant stakeholders (e.g., medical practitioners, policy makers, and researchers) an opportunity to better understand the needs and thoughts of target patients, to disseminate evidence-based information, and to formulate new research questions. For example, our analysis showed that some patients with spinal stenosis might have some suicidal thoughts, which has not been mentioned in prior research. Further, policymakers should recognize the negative physical and psychological impacts of spinal stenosis, and develop relevant policies to alleviate these impacts on patients. Our results also reveal that some academics chose to disseminate research findings, while many patients desire to read credible disease-specific information. This finding highlights that social media can be an inexpensive alternative platform to educate patients and conduct research globally. It may also be used to detect/monitor public health emergencies that may not be achieved by traditional data collection methods (e.g., questionnaires) [66]. That said, Twitter can only be an adjunct approach to supplement traditional methods in data collection and knowledge translation.

The current study has several limitations. First, it is impossible to know the detailed demographics of Twitter users [67]. The gender and occupational information were estimated based on artificial intelligence algorithm in Talkwalker, which may limit its generalizability. Second, there was a possible selection bias of younger Twitter users. It was estimated that only 0.3% of Twitter users in England were 60 years or older [68], whereas approximately 67.5% of Twitter users were ages between 16 and 22 years [68]. That said, our content analyses revealed that the physical and psychological impacts of spinal stenosis shared by Twitter users were comparable to those reported by patients with spinal stenosis in prior research [37, 38, 43]. It suggests that Twitter users tweeting about stenosis are a representative sample of such patients. Future studies can use specific search terms on the Talkwalker to identify specific age groups to evaluate the differential influences of spinal stenosis on people at different ages. Third, our findings cannot be generalized to all countries (e.g., China or low-income countries), where Twitter is unavailable for political or technical reasons. Future research should consider analyzing results from other social media (such as Maipo) or using traditional paper-based questionnaires to reach out to these populations. Fourth, the current study only analyzed tweets posted between 29 May 2019 and 24 June 2020. Future studies should analyze tweets over a longer period to improve the representativeness of findings and to monitor the trend of health information seeking on Twitter. That said, the current proof of concept study has laid the foundation for future health analytic research in various social media platforms. Fifth, the current study used a general term ‘spinal stenosis’ as the keyword to search for relevant tweets. However, the causes and progression of traumatic cervical spinal stenosis differ from those of degenerative lumbar spinal stenosis. Future studies should use more specific search terms to identify tweets from target populations for analysis.

## Conclusions

The content analysis of tweets reveals that at least for people who tweet about their spinal stenosis, the condition appears to have substantial negative impacts on physical and psychosocial wellbeing in people with the disease. Our results indicate that Twitter users use the platform to seek health information and assistance from others. Similarly, researchers and healthcare professionals use Twitter to disseminate information regarding spinal stenosis although the effectiveness of this dissemination approach remains unclear. Overall, Twitter can be a novel channel for researchers to understand the impact of different diseases on various aspects of patients, conduct research (e.g., online surveys), and disseminate research findings.

## Data Availability

Datasets used and/or analysed in this study can be obtained from the corresponding author upon reasonable request.
